# Long-term outcomes among Medicare patients readmitted in the first year of hemodialysis: a retrospective cohort study

**DOI:** 10.1186/s12882-019-1473-0

**Published:** 2019-07-29

**Authors:** Katherine H. Ross, Bernard G. Jaar, Janice P. Lea, Tahsin Masud, Rachel E. Patzer, Laura C. Plantinga

**Affiliations:** 1 Department of Epidemiology, Emory Rollins School of Public Health, Atlanta, GA USA; 2 Department of Medicine, Johns Hopkins School of Medicine, Baltimore, MD USA; 3 Welch Center for Prevention, Epidemiology and Clinical Research, Johns Hopkins University, Baltimore, MD USA; 4 Department of Epidemiology, Johns Hopkins Bloomberg School of Public Health, Baltimore, MD USA; 5 Nephrology Center of Maryland, Baltimore, MD USA; 6 Department of Medicine, Emory University School of Medicine, Atlanta, GA USA; 7 Department of Surgery, Emory University School of Medicine, Atlanta, GA USA

**Keywords:** Hemodialysis, Hospital readmissions, Mortality, Morbidity, Kidney transplantation

## Abstract

**Background:**

Readmission within 30 days of hospital discharge is common and costly among end-stage renal disease (ESRD) patients. Little is known about long-term outcomes after readmission. We estimated the association between hospital admissions and readmissions in the first year of dialysis and outcomes in the second year.

**Methods:**

Data on incident dialysis patients with Medicare coverage were obtained from the United States Renal Data System (USRDS). Readmission patterns were summarized as no admissions in the first year of dialysis (Admit-), at least one admission but no readmissions within 30 days (Admit+/Readmit-), and admissions with at least one readmission within 30 days (Admit+/Readmit+).We used Cox proportional hazards models to estimate the association between readmission pattern and mortality, hospitalization, and kidney transplantation, accounting for demographic and clinical covariates.

**Results:**

Among the 128,593 Medicare ESRD patients included in the study, 18.5% were Admit+/Readmit+, 30.5% were Admit+/Readmit-, and 51.0% were Admit-. Readmit+/Admit+ patients had substantially higher long-term risk of mortality (HR = 3.32 (95% CI, 3.21–3.44)), hospitalization (HR = 4.46 (95% CI, 4.36–4.56)), and lower likelihood of kidney transplantation (HR = 0.52 (95% CI, 0.44–0.62)) compared to Admit- patients; these associations were stronger than those among Admit+/Readmit- patients.

**Conclusions:**

Patients with readmissions in the first year of dialysis were at substantially higher risk of poor outcomes than either patients who had no admissions or patients who had hospital admissions but no readmissions. Identifying strategies to both prevent readmission and mitigate risk among patients who had a readmission may improve outcomes among this substantial, high-risk group of ESRD patients.

**Electronic supplementary material:**

The online version of this article (10.1186/s12882-019-1473-0) contains supplementary material, which is available to authorized users.

## Background

End-stage renal disease (ESRD) patients on hemodialysis experience a high burden of hospital admission and readmission. In 2015, ESRD patients were admitted to the hospital 1.7 times per year on average, and about 35% of hospital discharges among these patients were followed by a readmission within 30 days of discharge [1] – almost double the readmission rate of the general Medicare population. This contributes to the overall economic burden of ESRD, as approximately one-third of Medicare expenditures for ESRD patients are for inpatient costs [1]. The Centers for Medicare & Medicaid Services (CMS) have implemented quality metrics designed to incentivize reducing readmissions, including the standardized readmission ratio (SRR) for dialysis facilities. Several studies have focused on identifying predictors of readmission among ESRD patients, including demographics [2], comorbidities [3, 4], clinical characteristics of the hospital stay [5], psychosocial factors [6], and nephrology visits and other processes of care after initial hospitalization [7, 8]. However, less is known about health outcomes after readmission.

Readmission within 30 days is associated with 1-year mortality in the community-dwelling Medicare population [9], and we have previously demonstrated an association between readmission and 1-year mortality among prevalent ESRD patients [10]. The relationship between early readmission among incident ESRD patients and longer-term outcomes remains unexplored. Patients who have had an early readmission may represent a high-risk subgroup that have a different disease course and mortality risk than the already elevated risk experienced by ESRD patients [11]. Characterizing long-term outcomes among those who have had an early readmission may inform clinical decision-making and interventions to improve care for this substantial group. We used the United States Renal Data System (USRDS) to estimate the association between early readmission in the first year of dialysis and mortality, hospitalization, and transplantation in the second year among a cohort of incident hemodialysis patients who survived at least 1 year with primary Medicare coverage in the United States.

## Methods

### Study population and data sources

**Fig. 1:**
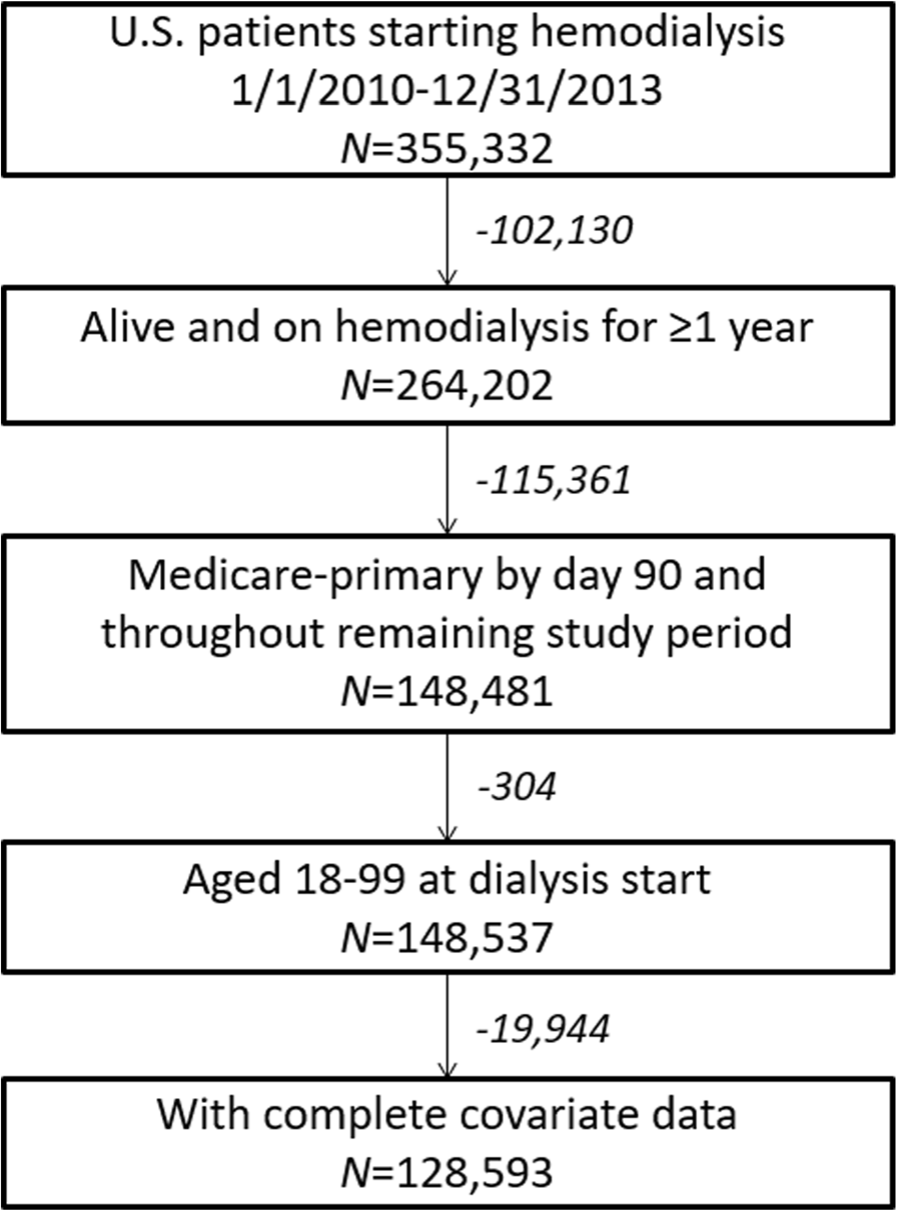
Flow diagram showing study sample selection among 2010–2013 incident U.S. hemodialysis patients

For this retrospective cohort study, we identified 264,202 patients who started in-center hemodialysis treatment between 1/2010 and 12/2013 and remained alive and on hemodialysis continuously for at least 1 year. Patients were excluded if they remained alive but did not have primary Medicare coverage over the entire study period (to avoid differential capture of index admissions and readmissions in the presence of secondary payor; *n* = 115,361), if they were < 18 or > 100 years old (*n* = 304), or if they were missing data on covariates (*n* = 19,944), leaving 128,593 patients in our analytic cohort (Fig. 1). Data, including hospitalization information and patient characteristics and outcomes, were obtained from the USRDS, and analyses were approved by the Emory Institutional Review Board.

### Study variables

#### Readmission pattern

**Fig. 2:**
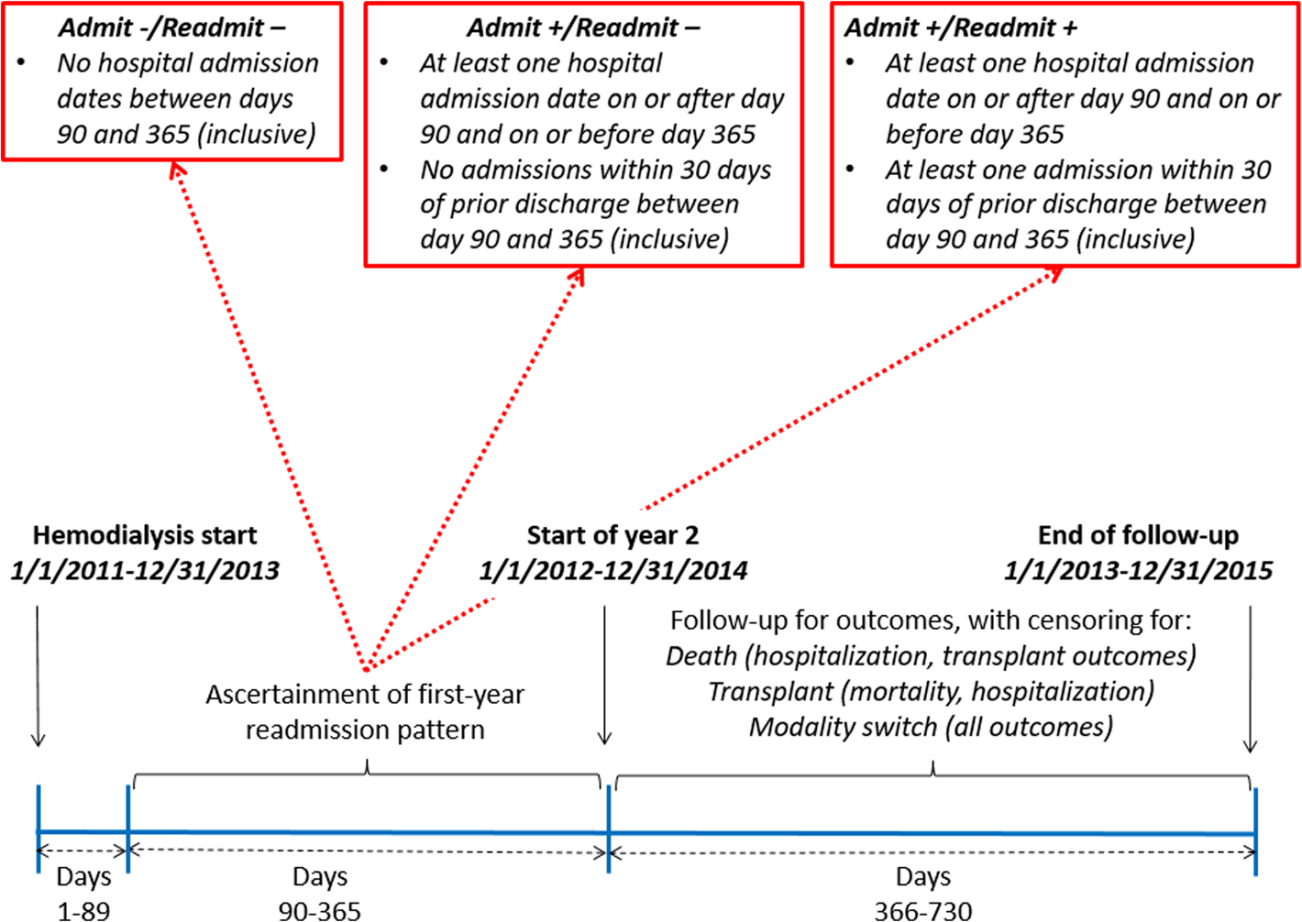
Timeline showing ascertainment of readmission pattern and outcomes in our cohort of 2010–2013 incident U.S. hemodialysis patients

Hospital admissions between day 90 and day 365 of hemodialysis treatment were included as first-year hospitalizations. This 90-day lag period was included to account for lags in Medicare coverage among incident hemodialysis patients. Readmissions were defined as admissions within 30 days of discharge from the previous hospitalization. First-year readmission patterns were defined as follows: “no admissions (Admit-)” if no hospital admissions occurred during days 90–365 of hemodialysis; “admissions, no readmissions (Admit+ / Readmit-)” if at least one hospital admission, but no readmissions, occurred during days 90–365; and “readmissions (Admit+ / Readmit+)” if at least one hospital readmission within 30 days of index hospitalization discharge occurred during days 90–365. Figure 2 describes the timing of exposure ascertainment.

#### Outcomes

Outcomes were defined in the second year of treatment (days 366–730). The follow-up period was additionally censored at death, receipt of a kidney transplant, switch to peritoneal dialysis, or loss to follow-up, as appropriate for the outcome. Death, receipt of first kidney transplant, and first hospitalization were treated as time-to-event outcomes (follow-up = date of event – date of day 366). Counts of all hospitalizations were examined as rates (counts of hospitalizations per follow-up, excluding time spent in the hospital).

#### Other variables

Patient age at dialysis initiation, sex, race/ethnicity, receipt of pre-ESRD nephrology care, vascular access used at dialysis initiation, insurance at dialysis initiation, and presence/history of comorbid conditions at dialysis initiation were obtained from CMS Form 2728 data available from USRDS. Intensive care unit (ICU) utilization during hospitalizations was obtained from hospital data files.

### Statistical analysis

Patient characteristics were summarized overall and by first-year readmission pattern. Distributions of outcomes in the second year of dialysis were plotted according to first-year readmission patterns. Kaplan-Meier curves were used to examine crude associations of first-year readmission pattern with time-to-event outcomes. Multivariable Cox proportional hazards models were used to estimate the associations of readmission pattern with mortality, first hospitalization, and kidney transplant (hazard ratios) and multivariable negative binomial models were used to estimate associations of readmission pattern with hospitalization rates (incidence rate ratios), along with 95% confidence intervals. Of a potential list of confounding variables, we included in the final model those which were associated with a change in the associations of other variables in the model with readmission (i.e., substantial confounding effect) and/or those which we considered a priori to be confounders. Secondarily, we examined ICU utilization in second-year hospitalizations. We performed a complete case analysis of all data. Analyses were performed with Stata v. 14.2 (College Station, TX).

### Sensitivity analyses

We performed several sensitivity analyses to assess the robustness of our results to various assumptions. First, we restricted the cohort to those who had primary Medicare coverage from the first day of dialysis in order to determine whether the 90-day run-in period induced bias. Second, we defined comorbid conditions as being present if it was either indicated on CMS Form 2728 *or* in the Medicare claims data, defined from discharge codes from all hospital discharges in the first year of dialysis, using the diagnostic codes outlined in the CMS Chronic Conditions Warehouse algorithms [12]. Next, we re-defined the readmissions pattern so that emergency department (ED) or observational stay was considered to be a hospital admission. In addition, we also evaluated readmission patterns by cause of hospital admission for cardiovascular (CV) and infectious disease (ID)-related admissions. CV-related admissions were classified as followed: no CV admission (CV Admit-), a CV-related admission but no CV-related readmission (CV Admit+/Readmit-) or a CV-related admission and a CV-related readmission within 30 days (CV Admit+/Readmit+). ID-related admissions were classified as follows: no ID admission (ID Admit-), an ID-related admission but no ID-related readmission (ID Admit+/Readmit-) or an ID-related admission and a ID-related readmission within 30 days (ID Admit+/Readmit+). Cause of admission (cardiovascular and infectious) was determined by the primary discharge code, using ICD-9 codes as defined by the USRDS [1]. Finally, we performed Fine and Gray regression to account for mortality as a competing risk [13] for time to readmission and time to transplant.

## Results

### Patient characteristics by first-year readmission pattern

Among the 128,593 Medicare patients included in the study, 18.5% were Admit+ / Readmit+, while 30.5% were Admit+ / Readmit-, and about half (51.0%) were Admit- in the first year (Table 1). The mean age of the cohort was 64.2 years, 43.8% of patients were female, and 30.9% were black. Most of the patients at index admission had hypertension (90.4%) and diabetes (60.4%). Those who were Admit+ / Readmit+ were younger (63.3 years) than those who were Admit+ / Readmit- (65.6 years) or Admit- (64.3 years); they were also more likely to be female and non-Hispanic white (Table 1). Patients who were Admit+ / Readmit+ were also more likely to be functionally impaired or to have diabetes, congestive heart failure, peripheral vascular disease, cerebrovascular disease, atherosclerotic heart disease, and chronic obstructive pulmonary disease, compared to those who were Admit+ / Readmit- and, particularly, those who were Admit-. Indicators of better access to care, including receipt of pre-ESRD care, vascular access used on first dialysis, and private insurance type, were all less frequent in patients who were Admit+ / Readmit+ in the first year (Table 1).

**Table 1:** Selected patient characteristics of 2010–2013 incident U.S. hemodialysis patients, overall and by readmission pattern in the first 90–365 days of dialysis

Characteristic	Overall	First-year readmission pattern		
		No admissions (Admit-)	Admissions, no readmissions (Admit+/Readmit-)	Readmissions (Admit+/Readmit+)
*N (%)*	*128,593 (100%)*	*65,629 (51.0%)*	*39,266 (30.5%)*	*23,698 (18.5%)*
Patient demographics				
Mean (SD) age, years	64.17 (14.51)	64.25 (14.39)	65.59 (14.51)	63.26 (14.78)
Sex (%)				
Female	43.82%	40.46%	46.36%	48.91%
Male	56.18%	59.54%	53.64%	51.09%
Race/ethnicity (%)				
Non-Hispanic white	48.67%	47.14%	50.14%	50.46%
Black	30.93%	31.32%	30.19%	31.09%
Hispanic	14.13%	14.37%	14.18%	13.39%
Other	6.27%	7.17%	5.50%	5.06%
Comorbid conditions at dialysis start				
Diabetes (%)	60.36%	57.75%	61.87%	65.07%
Hypertension (%)	90.37%	91.02%	98.71%	89.67%
Congestive heart failure (%)	32.53%	29.02%	35.11%	37.99%
Cerebrovascular disease (%)	10.15%	9.06%	10.94%	11.84%
Atherosclerotic heart disease (%)	20.47%	18.72%	21.97%	22.85%
Peripheral vascular disease (%)	15.05%	12.81%	16.41%	19.03%
Cancer (%)	6.86%	6.65%	7.15%	6.95%
COPD (%)	9.79%	8.01%	11.05%	12.64%
Functional impairment^a^ (%)	16.56%	13.82%	18.31%	21.25%
Access to care				
Received pre-ESRD nephrology care (%)	69.34%	71.49%	68.37%	64.98%
Vascular access used on first dialysis (%)				
Fistula	20.90%	25.54%	17.51%	13.69%
Graft	3.72%	3.95%	3.61%	3.25%
Catheter	75.38%	70.50%	78.89%	83.06%
Insurance at dialysis start (%)				
Private	9.97%	10.71%	9.37%	8.90%
Medicare	24.57%	23.89%	25.10%	25.61%
Medicaid	31.34%	28.73%	32.66%	36.40%
Other	23.86%	25.57%	23.24%	20.16%
None	10.26%	11.11%	9.64%	8.93%

*P* < 0.001 for all comparisons by ANOVA or chi-square, as appropriate

*COPD* Chronic obstructive pulmonary disease, *ESRD* End-stage renal disease

^a^Functional impairment defined by needing assistance with at least one activity of daily living, inability to walk, inability to transfer, or being institutionalized

### Association of first-year readmission pattern with outcomes

#### Mortality

**Fig. 3:**
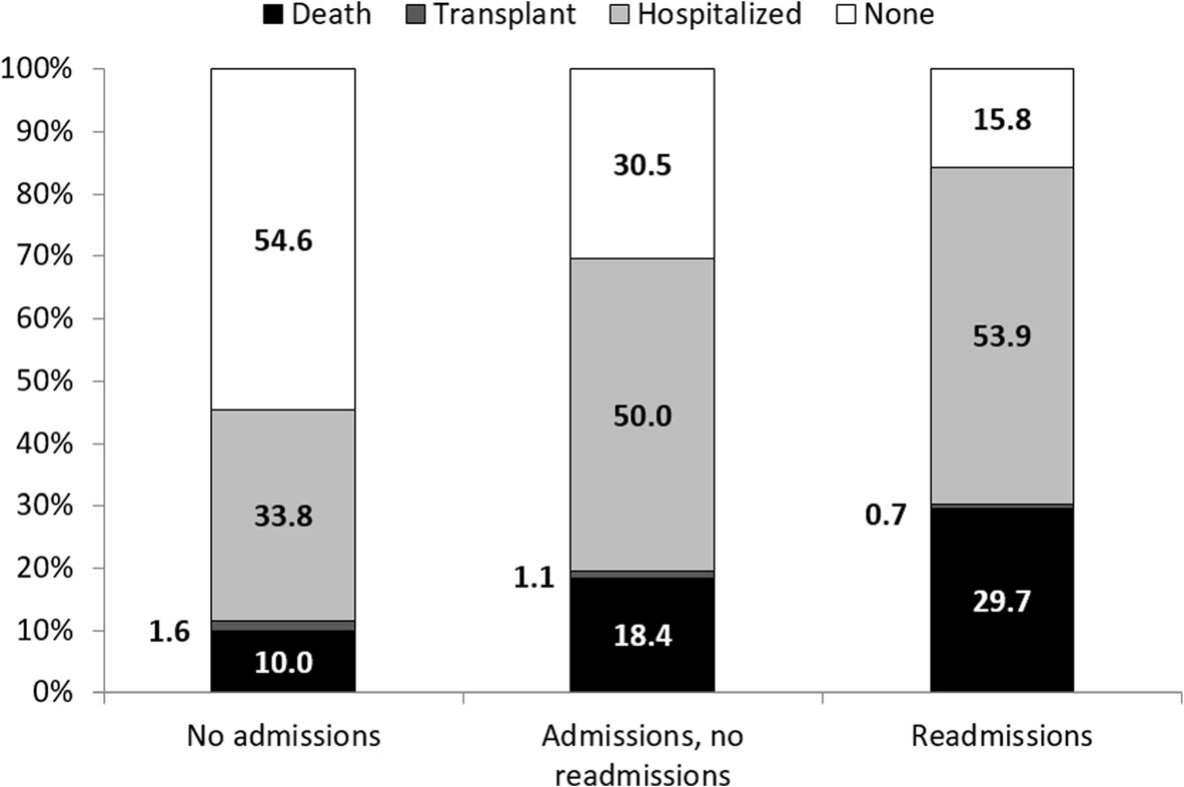
Distributions of outcomes in the second year of dialysis treatment among 2010–2013 incident U.S. hemodialysis patients by readmission pattern in the first year of dialysis. Hospitalized = admitted at any point in year 2 but remaining alive and on dialysis during year 2

**Fig 4:**
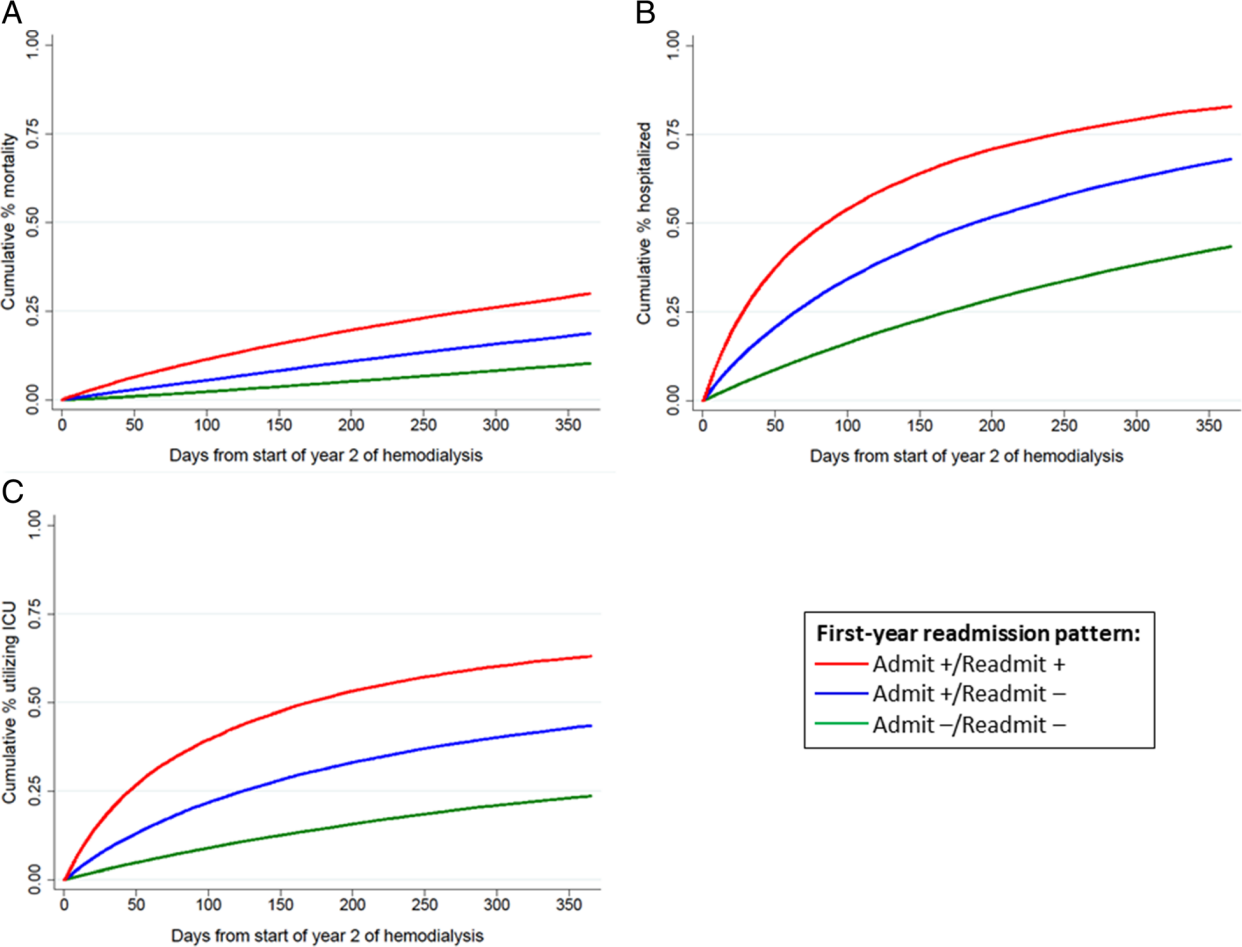
Cumulative mortality (**a**), hospitalization (**b**), and hospitalization involving intensive care unit (ICU) utilization (**c**) in second year of hemodialysis by readmission pattern in their first year of dialysis, among 2010–2013 incident U.S. hemodialysis patients. *P* < 0.001 by log-rank for all

Cumulative mortality risk in year 2 among those who were Admit-, Admit+ / Readmit-, and Admit+ / Readmit+ was 10.0, 18.4, and 29.7%, respectively (Figs. 3 and 4a). Crude analyses showed that, compared to patients who were Admit- in the first year of hemodialysis, those who were Admit+ / Readmit- and those who were Admit+ / Readmit+ were 1.9- and 3.4-fold, respectively, more likely to die in the second year of hemodialysis (Fig. 4a, Table 2). With adjustment for demographics and comorbid conditions (congestive heart failure, diabetes, and hypertension), these patients remained at 1.9- and 3.5-fold higher risk. Further adjustment for access to care indicators did not substantially change these results (Table 2), nor did additional adjustment for atherosclerotic heart disease, peripheral vascular disease, cerebrovascular disease, chronic obstructive pulmonary disease, cancer, and functional impairment [Admit+ / Readmit-: HR = 1.83 (95% CI, 1.77–1.89); Admit+ / Readmit+: HR = 3.39 (95% CI, 3.28–3.50)].

**Table 2:** Associations of second-year outcomes with hospital admission pattern in the first 90–365 days of dialysis, among 2010–2013 U.S. incident hemodialysis patients

Outcome	First-year readmission pattern		
	No admissions (Admit-)	Admissions, no readmissions (Admit+/Readmit-)	Readmissions (Admit+/Readmit+)
Mortality, hazard ratio (95% CI)			
Crude rate per 1000 patient-years	107.1	206.9	362.3
Unadjusted	1.00 (ref.)	1.93 (1.87–2.00)	3.38 (3.27–3.50)
Adjusted^a^	1.00 (ref.)	1.89 (1.83–1.96)	3.48 (3.36–3.60)
Adjusted^a^ + access to care	1.00 (ref.)	1.84 (1.78–1.90)	3.32 (3.21–3.44)
Hospitalization, incidence rate ratio (95% CI)			
Crude rate per 1000 patient-years	966.6	2051.7	3922.2
Unadjusted	1.00 (ref.)	2.26 (2.22–2.30)	4.75 (4.64–4.85)
Adjusted^a^	1.00 (ref.)	2.21 (2.17–2.26)	4.62 (4.53–4.73)
Adjusted^a^ + access to care	1.00 (ref.)	2.16 (2.13–2.21)	4.46 (4.36–4.56)
Transplantation, hazard ratio (95% CI)			
Crude rates per 1000 patient-years	17.30	13.10	8.44
Unadjusted	1.00 (ref.)	0.74 (0.67–0.83)	0.46 (0.39–0.55)
Adjusted^a^	1.00 (ref.)	0.78 (0.70, 0.87)	0.46 (0.39–0.54)
Adjusted^a^ + access to care	1.00 (ref.)	0.85 (0.76–0.95)	0.52 (0.44–0.62)

^a^Adjusted for age at dialysis start, sex, race/ethnicity, and comorbid conditions at dialysis start (congestive heart failure, diabetes, and hypertension). Access to care includes pre-ESRD nephrology care, vascular access on first dialysis, and insurance at dialysis start

#### Hospitalization

Among patients who survived the first year and remained on hemodialysis in year 2, 33.8, 50.0, and 53.9% of those who were Admit-, Admit+ / Readmit-, and Admit+ / Readmit+ in year 1, respectively, were hospitalized at least once in year 2 (Fig. 3). Among year 2 admissions in patients who were Admit-, Admit+ / Readmit- and Admit+ / Readmit+ in year 1, 43.6, 48.1, and 57.0%, respectively, involved ICU utilization.

Time to first hospitalization was significantly lower among those who were Admit+ / Readmit+ and Admit+ / Readmit-, compared to those who were Admit- (Fig. 4b), and even with adjustment for demographic and clinical characteristics, these patients were 2.0- and 3.3-fold more likely to be admitted [Admit+ / Readmit-: HR = 1.97 (95% CI, 1.94–2.00); Admit+ / Readmit+: HR = 3.27 (95% CI, 3.21–3.33)]. Time to first hospitalization with ICU utilization was also significantly lower among those who were Admit+ / Readmit+ and Admit+ / Readmit- compared to those without first-year admissions (Fig. 4c), and this association was also robust to adjustment [Admit+ / Readmit-: HR = 2.14 (95% CI, 2.09–2.20); Admit+ / Readmit+: HR = 4.04 (95% CI, 3.95–4.15)].

Hospitalization rates (including all admissions in year 2 over follow-up) were 2.3 and 5.0 times higher among those who were Admit+ / Readmit- and those who were Admit+ / Readmit+ in year 1, compared to those who were Admit- (Table 2). Associations were not substantially changed with adjustment, including adjustment for access to care indicators (Table 2). Adjustment for additional comorbid conditions also did not change the results. [Admit+ / Readmit-: HR = 2.16 (95% CI, 2.13–2.21); Admit+ / Readmit+: HR = 4.46 (95% CI, 4.36–4.56)].

#### Transplantation

Among incident hemodialysis patients who were Admit-, Admit+ / Readmit-, and Admit+ / Readmit+ in the first year, 1.6, 1.1, and 0.7% were transplanted in the second year (Fig. 3). Kaplan-Meier analysis of time to transplant suggested cumulative incidence of transplant in year 2 differed significantly by readmission pattern in year 1 (*P* < 0.001 by log-rank). Those who were Admit+ / Readmit- and those who were Admit+ / Readmit+ were 26 and 54% less likely, respectively, than those who were Admit- to be transplanted (Table 2); with adjustment for demographics and comorbid conditions (congestive heart failure, diabetes, and hypertension), these patients were 22 and 54% less likely to be transplanted. Further adjustment for access to care indicators attenuated these associations somewhat (Table 2); adjustment for additional comorbid conditions (atherosclerotic heart disease, peripheral vascular disease, cerebrovascular disease, chronic obstructive pulmonary disease, cancer, and functional impairment) showed similar results as well [Admit+ / Readmit-: HR = 0.85 (95% CI, 0.76–0.95); Admit+ / Readmit+: HR = 0.52 (95% CI, 0.44–0.62)].

#### Sensitivity analyses

We conducted several sensitivity analyses to assess the robustness of our results (Additional file 1: Table S1). First, we performed our analyses in a cohort of those who had Medicare as their primary insurance from day 1, and found that results were similar across all outcomes (mortality, readmission rate, time to first readmission in the second year, and time to transplant). Similarly, we performed analyses using Medicare claims and CMS Form 2728 to determine comorbidity, and found similar results across all outcomes. We found that results for all outcomes were attenuated towards the null when we incorporated ED/observation stays into readmission patterns, and that results were similar for both causes of initial admission that we considered (CVD and ID). Finally, we performed analyses on time to hospital readmission and time to transplant considering mortality as a competing risk (Additional file 2: Table S2), and found that the sub-distribution hazard ratios were slightly attenuated towards the null compared to the hazard ratios from the original analysis for time to readmission, but were further from the null for time to transplant.

## Discussion

In this analysis of the U.S. Medicare population initiating hemodialysis in 2010–2013 who survived at least 1 year on dialysis, nearly 1 in 5 experienced a hospital admission with 30-day readmission in the first year of hemodialysis. We found that 30-day readmissions were associated with increased risk of subsequent long-term outcomes in the second year of hemodialysis, including mortality, hospitalization, ICU utilization, and a lower likelihood of kidney transplantation. Patients with readmissions were at substantially higher risk of poor outcomes than either patients who had no admissions in the first year or patients who had a hospital admission with no readmission. Our findings imply that the detrimental effects of readmission are long-lasting and affect a large proportion of incident dialysis patients. To our knowledge, this is the first study to identify patients with hospital readmissions in their first year of dialysis as a group at high risk for poor long-term outcomes.

Our observed associations likely have complex mechanisms. One possible explanation for our observed association between hospital readmission and subsequent poor outcomes is that hospital readmission is a marker for patients who have a poor long-term prognosis. We found patients with readmissions in the first year of dialysis had higher rates of comorbidities than patients who were either not admitted or admitted but not readmitted. Chan, et al. [3], also found that patients who were readmitted had higher rates of comorbidities, as well as more severe disease. While we adjusted for the presence of comorbidities in multivariable analyses, and performed additional sensitivity analyses using comorbidities from claims as well as the 2728 form, we did not adjust for all possible diagnoses, or for comorbidity severity. Therefore, there is likely residual confounding of our results by underlying patient health status.

Another potential explanation for our findings is that physiologic changes in ESRD patients during hospitalization contribute to poor long-term outcomes. Chan et al. found decreased levels of hemoglobin, albumin, phosphorus, calcium, parathyroid hormone and weight after hospitalization among dialysis patients; these lab values were inversely correlated with length of stay [14]. The authors suggested that hospitalization could be described as an “acute inflammation-malnutrition syndrome” which impacts laboratory markers associated with mortality in ESRD. Readmissions result in increased time spent in the hospital, potentially magnifying the effect of malnutrition. Increased time spent in the hospital may also increase the risk of hospital-acquired infections, which are a major cause of mortality among ESRD patients [1]. It is unclear from our study - or other previous studies - whether hospitalization or readmission events have a causal effect on long-term outcomes, or whether they are markers of poor prognosis, or both. The most effective approach to improving outcomes for these patients likely involves both attempts to prevent readmission and interventions to reduce mortality risk for patients who have been readmitted.

Patients who were Admit+ / Readmit+ in our study were less likely than those who were Admit-, or Admit+ / Readmit-, to receive a kidney transplant. Kidney transplantation is the preferred treatment for ESRD, conferring improved survival and quality of life to patients as well as decreased healthcare costs [15]. However, not all patients benefit equally from transplant. A study by Lynch et al., found that patients who were frequently admitted to the hospital while on the waitlist for kidney transplant had increased waitlist mortality, increased healthcare utilization, and inferior graft and overall survival [16]. Simply increasing access to transplantation among those with readmissions may not result in substantially improved outcomes without a focus on improving their underlying health status.

The readmissions rate in our study is lower than the USRDS estimate of 35% among prevalent patients; our analysis was at the patient-level, as opposed to the admissions-level, and focused only on incident hemodialysis patients who survived at least 1 year. Our observed rate of 19% was slightly lower than both the rates we reported in a previous study of the Medicare population (23%) [10] and a study by Chan, et al., that included the non-Medicare population (22%) [3]; this difference may be due to the fact that patients must have survived at least 1 year to be included in our cohort. While no other studies have directly examined the association between readmissions and long-term mortality, our estimated effect of hospital admissions alone on mortality (HR: 1.84, 95% CI: 1.78–1.90) was comparable to that observed in a study of long-term mortality among ESRD patients after ICU admission (HR: 2.32, 95% CI: 1.84–2.92) [17].

Our results should be interpreted in the context of several limitations not mentioned above. First, there is the potential for misclassification of variables in administrative databases such as USRDS. Second, we did not have access to laboratory data during follow up, which may have provided insight into preliminary markers of poor outcomes. Third, our assessment of hospital admissions and readmissions began at 90 days after dialysis initiation; patients may have had hospital admissions prior to this 90-day window that were not captured in our assessment. However, our primary purpose was to assess the association of outcomes subsequent to readmissions after a period of stabilization following dialysis initiation, and sensitivity analyses examining readmissions from day 1 showed similar results to our primary analyses. Additionally, under CMS' Hospital Readmissions Reduction Program hospitals are incentivized to avoid readmission by keeping patients in the ED or in observation stays, and we did not include these encounters in our primary analysis. In fact, results from our sensitivity analyses including ED visits/observation stays suggest that the sicker patients with the worst outcomes are less likely to be kept in the ED and instead admitted. However, it is important to note that our results reflect the actual outcomes subsequent to readmissions, as they are occurring under current policies. Finally, although we controlled for many relevant covariates, there is the possibility of residual confounding by factors not well-captured in our data, such as socioeconomic status, laboratory data, and severity of comorbid conditions. One major strength of our analysis is the use of USRDS, a national database of incident dialysis patients on Medicare. As the vast majority of ESRD patients in the United States are eligible for Medicare, our results are likely to be generalizable to the population of U.S. hemodialysis patients.

While potential interventions have been identified to prevent readmissions after hospitalization among ESRD patients – for example, additional physician visits [7] or hemoglobin monitoring [14] – very little is known about preventing poor outcomes among patients once they have had a readmission. Further studies are needed to identify modifiable factors associated with poor long-term outcomes for ESRD patients with hospital readmissions in their first year on dialysis, who represent about 20% of patients starting hemodialysis. Identification of these modifiable factors is the first step to clarifying potential interventions and directing appropriate clinical resources to this high-risk group.

## Conclusions

Hospital readmissions are common in the first year on hemodialysis and associated with an increased risk of poor long-term outcomes, including mortality, hospitalization, ICU utilization and lower likelihood kidney transplant. Identifying strategies to both prevent readmission and mitigate risk among patients who had a readmission may improve clinical care and outcomes among this substantial and high-risk group of ESRD patients.

## Additional files

Additional file 1: **Table S1.** Results of sensitivity analyses for the association of readmission pattern with mortality, readmission rate, time to first readmission, and time to transplant. (DOCX 14 kb)

Additional file 2: **Table S2.** Results of Fine and Gray regression for the association of time to readmission and time to transplant, accounting for mortality as a competing risk. (DOCX 14 kb)

## Data Availability

The data that support the findings of this study are available from the United States Renal Data System but restrictions apply to the availability of these data, which were used under license for the current study, and so are not publicly available. Data are however available from the authors upon reasonable request and with permission of USRDS.

## Abbreviations

CMS: Centers for Medicare & Medicaid Services
CV: Cardiovascular
ED: Emergency department
ESRD: End-stage renal disease
HR: Hazard ratio
ICU: Intensive care unit
ID: Infectious disease
SRR: Standardized readmissions ratio
USRDS: United States Renal Data System
