# Supplementary material for: Long-term outcomes among Medicare patients readmitted in the first year of hemodialysis: a retrospective cohort study
Source: BMC Nephrol. 2019 Jul 29;20:285. doi: 10.1186/s12882-019-1473-0 (PMC6664786; doi:10.1186/s12882-019-1473-0)
Supplement: Supplementary file 1 — Table S1. Results of sensitivity analyses for the association of readmission pattern with mortality, readmission rate, time to first readmission, and time to transplant. (DOCX 14 kb) [file 12882_2019_1473_MOESM1_ESM.docx]

**Table S1.** Results of sensitivity analyses for the association of readmission pattern with mortality, readmission rate, time to first readmission, and time to transplant.

|  | Original Analyses | | Medicare from Day 1 | | Claims-Based Comorbidity | | ED/Observation Stays | | CVD Admissions | | ID Admissions | |
| --- | --- | --- | --- | --- | --- | --- | --- | --- | --- | --- | --- | --- |
|  | Admit+/ Readmit- | Admit+/ Readmit+ | Admit+/ Readmit- | Admit+/ Readmit+ | Admit+/ Readmit- | Admit+/ Readmit+ | Admit+/ Readmit- | Admit+/ Readmit+ | Admit+/ Readmit- | Admit+/ Readmit+ | Admit+/ Readmit- | Admit+/ Readmit+ |
| Mortality (HR, 95% CI) | 1.89 (1.83, 1.96) | 3.48 (3.36, 3.60) | 1.89 (1.83, 1.96) | 3.49 (3.38, 3.62) | 1.81 (1.75, 1.88) | 3.20 (3.09, 3.32) | 1.46 (1.39, 1.53) | 2.77 (2.67, 2.87) | 1.92 (1.86, 1.98) | 2.89 (2.74, 3.05) | 1.98 (1.92, 2.05) | 3.11 (2.94, 3.29) |
| Number of readmissions (IRR, 95% CI) | 2.21 (2.26, 4.73) | 4.63 (4.53, 4.73) | 2.22 (2.18, 2.27) | 4.66 (4.56, 4.77) | 2.11 (2.07, 2.16) | 4.23 (4.13, 4.33) | 1.61 (1.56, 1.65) | 3.52 (3.45, 3.59) | 2.25 (2.19, 2.30) | 3.83 (3.66, 4.01) | 2.18 (2.12, 2.23) | 3.31 (3.15, 3.47) |
| Time to first admission in second year (HR, 95% CI) | 2.00 (1.97, 2.04) | 3.35 (3.29, 3.42) | 2.01 (1.98, 2.05) | 3.37 (3.30, 3.43) | 1.92 (1.89, 1.96) | 3.12 (3.07, 3.19) | 1.51 (1.48, 1.55) | 2.68 (2.63, 2.73) | 1.99 (1.95, 2.02) | 2.94 (2.85, 3.04) | 1.92 (1.89, 1.96) | 2.75 (2.66, 2.85) |
| Time to transplant (HR, 95% CI) | 0.78 (0.70, 0.87) | 0.46 (0.39, 0.54) | 0.78 (0.70, 0.88) | 0.46 (0.39, 0.54) | 0.87 (0.78, 0.98) | 0.57 (0.48, 0.68) | 1.03 (0.91, 1.17) | 0.61 (0.55, 0.68) | 0.79 (0.68, 0.92) | 0.36 (0.23, 0.56) | 0.64 (0.55, 0.74) | 0.25 (0.15, 0.42) |

ED, emergency department; CVD, cardiovascular disease; ID, infectious disease. All results are from fully adjusted models, including age at dialysis start, sex, race/ethnicity, and comorbid conditions at dialysis start (congestive heart failure, diabetes, and hypertension).
