# Supplementary material for: Long-term outcomes among Medicare patients readmitted in the first year of hemodialysis: a retrospective cohort study
Source: BMC Nephrol. 2019 Jul 29;20:285. doi: 10.1186/s12882-019-1473-0 (PMC6664786; doi:10.1186/s12882-019-1473-0)
Supplement: Supplementary file 2 — Table S2. Results of Fine and Gray regression for the association of time to readmission and time to transplant, accounting for mortality as a competing risk. (DOCX 14 kb) [file 12882_2019_1473_MOESM2_ESM.docx]

**Table S2**. Results of Fine & Gray regression for the association of time to readmission and time to transplant, accounting for mortality as a competing risk.

|  | Original Cohort, Hazard Ratios (95% CIs) | | Competing Risks,  Subhazard Ratios (95% CIs) | |
| --- | --- | --- | --- | --- |
|  | Admit + / Readmit - | Admit + / Readmit + | Admit + / Readmit - | Admit + / Readmit + |
| Time to first admission in second year (HR, 95% CI) | 2.00 (1.97, 2.04) | 3.35 (3.29, 3.42) | 1.96 (1.93, 1.99) | 3.11 (3.05, 3.17) |
| Time to transplant (HR, 95% CI) | 0.78 (0.70, 0.87) | 0.46 (0.39, 0.54) | 0.71 (0.67, 0.74) | 0.38 (0.35, 0.41) |

All results are from fully adjusted models, including age at dialysis start, sex, race/ethnicity, and comorbid conditions at dialysis start (congestive heart failure, diabetes, and hypertension).
